# Supplementary material for: Anithiactin D, a Phenylthiazole Natural Product from Mudflat-Derived Streptomyces sp., Suppresses Motility of Cancer Cells
Source: Mar Drugs. 2024 Feb 14;22(2):88. doi: 10.3390/md22020088 (PMC10889970; doi:10.3390/md22020088)
Supplement: Supplementary file 1 [file marinedrugs-22-00088-s001.zip › marinedrugs-2849389-supplementary.pdf]

Supporting Information

Anithiactin D, a Phenylthiazole Natural Product from Mudflat-Derived *Streptomyces* sp., Suppresses Motility of Cancer Cells

Sultan Pulat [1,†], Inho Yang [2,†], Jihye Lee [3,4,†], Sunghoon Hwang [5], Rui Zhou [1], Chathurika D. B. Gamage [1], Mücahit Varlı [1], İsa Taş [1], Yi Yang [1], So-Yeon Park [1], Areum Hong [4], Jeong-Hyeon Kim [4], Dong-Chan Oh [5], Hangun Kim [1,*], Sang-Jip Nam [4,*] and Heonjoong Kang [6,*]

List of Contents

Figure S1. ^{1}H NMR spectrum (700 MHz, CD_3OD) of anthiactin D (**1**) **S2**

Figure S2. ^{13}C NMR spectrum (175 MHz, CD_3OD) of anthiactin D (**1**) **S3**

Figure S3. COSY spectrum (700 MHz, CD_3OD) of anthiactin D (**1**) **S4**

Figure S4. HSQC spectrum (700 MHz, CD_3OD) of anthiactin D (**1**) **S5**

Figure S5. HMBC spectrum (700 MHz, CD_3OD) of anthiactin D (**1**) **S6**

Figure S6. Comparing ECD spectrum with conformational search models of anthiactin D (**1**)........ **S7**

Figure S1. ^{1}H NMR spectrum (700 MHz, CD_3OD) of anthiactin D (**1**)

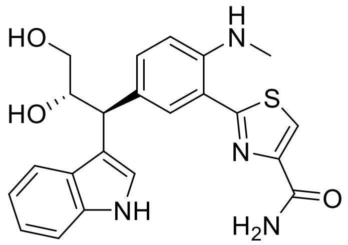

1

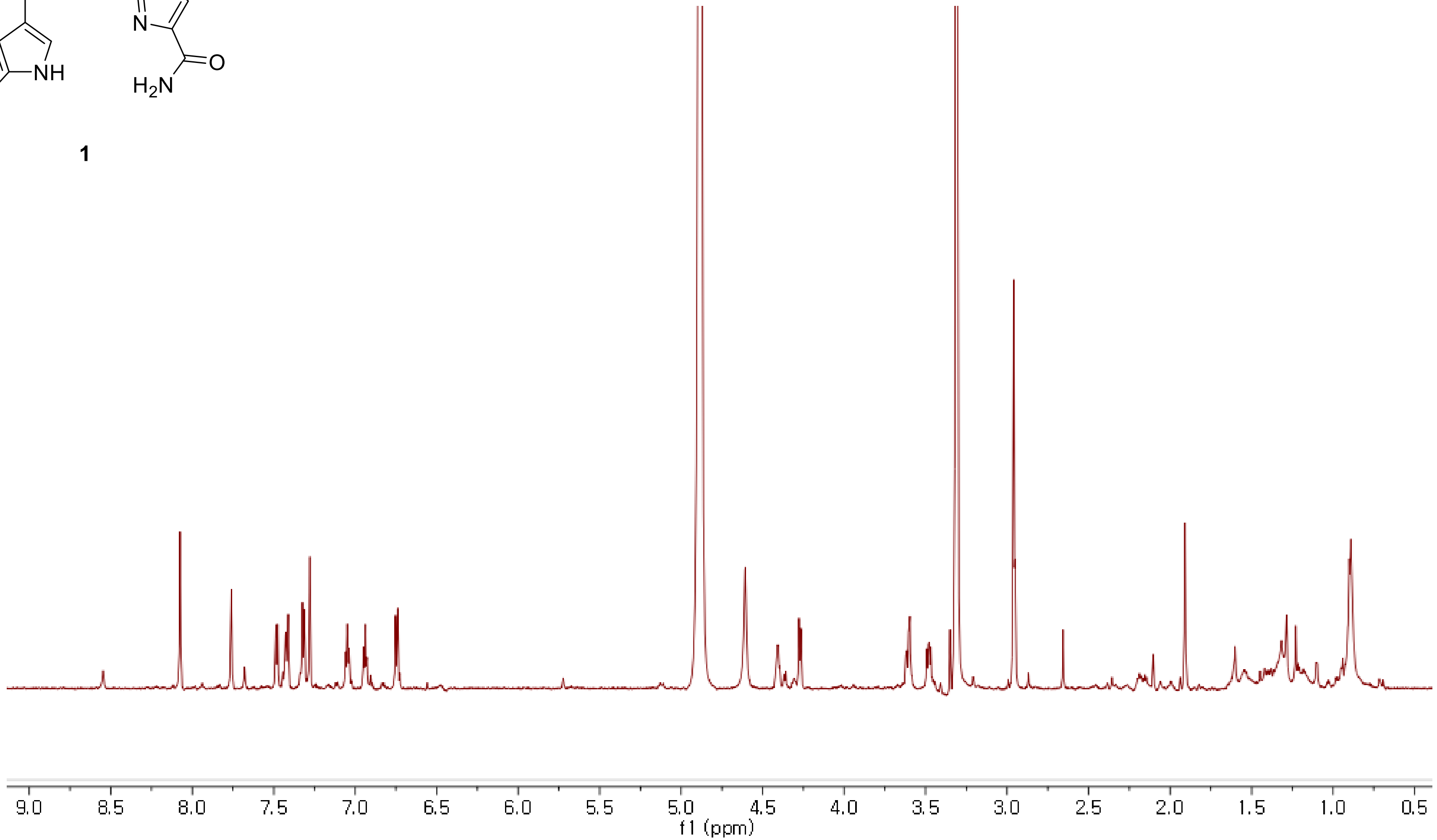

Figure S2. ^{13}C NMR spectrum NMR Spectrum (CD_3OD, 175 MHz) of anithiactin D (**1**)

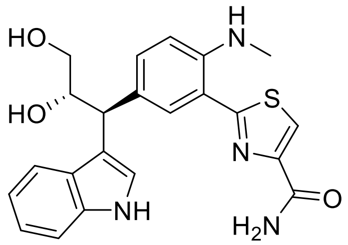

1

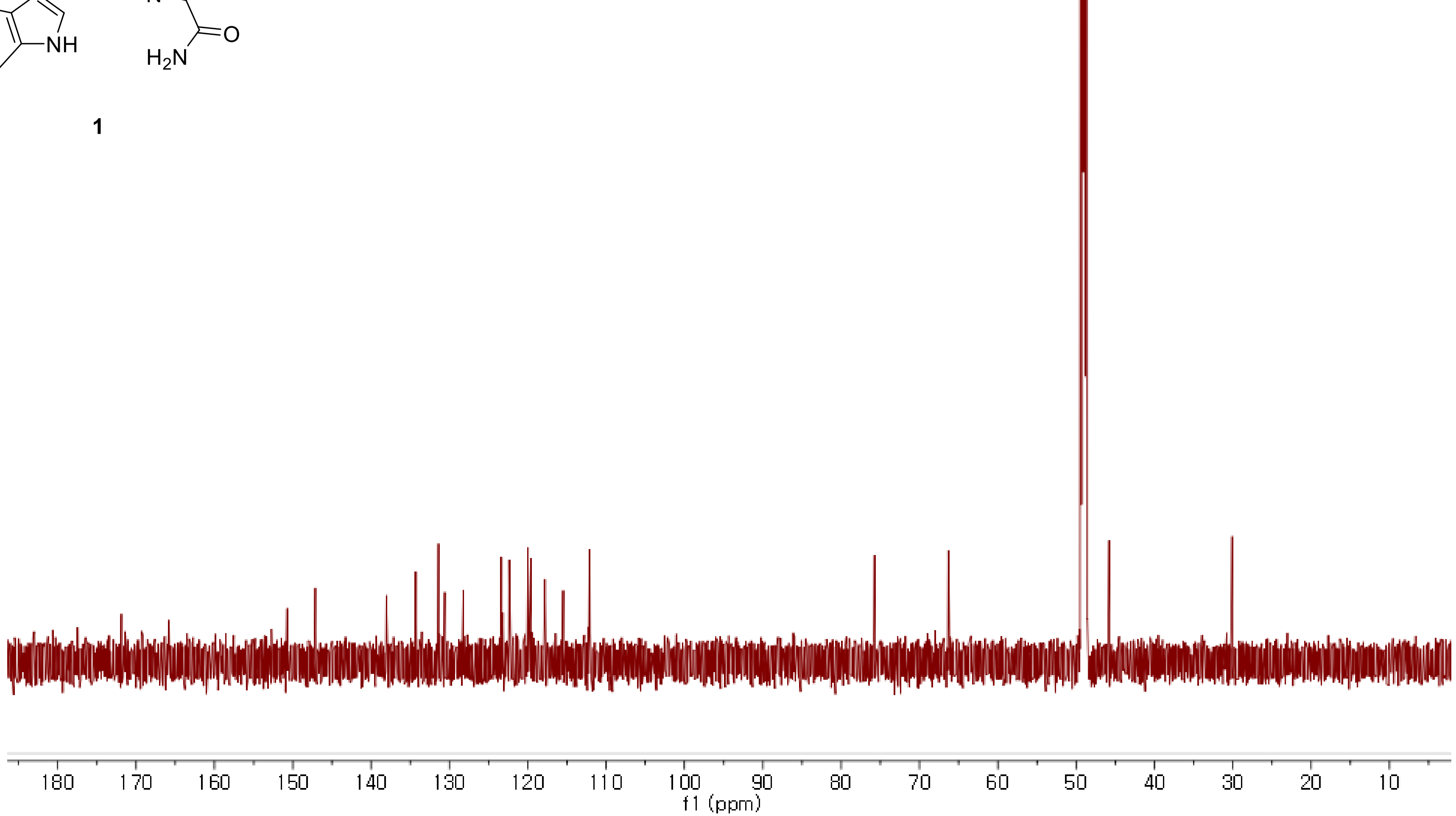

Figure S3. COSY NMR Spectrum (CD_3OD, 700 MHz) of anithiactin D (**1**)

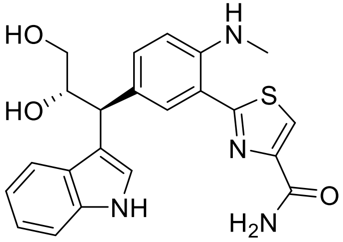

1

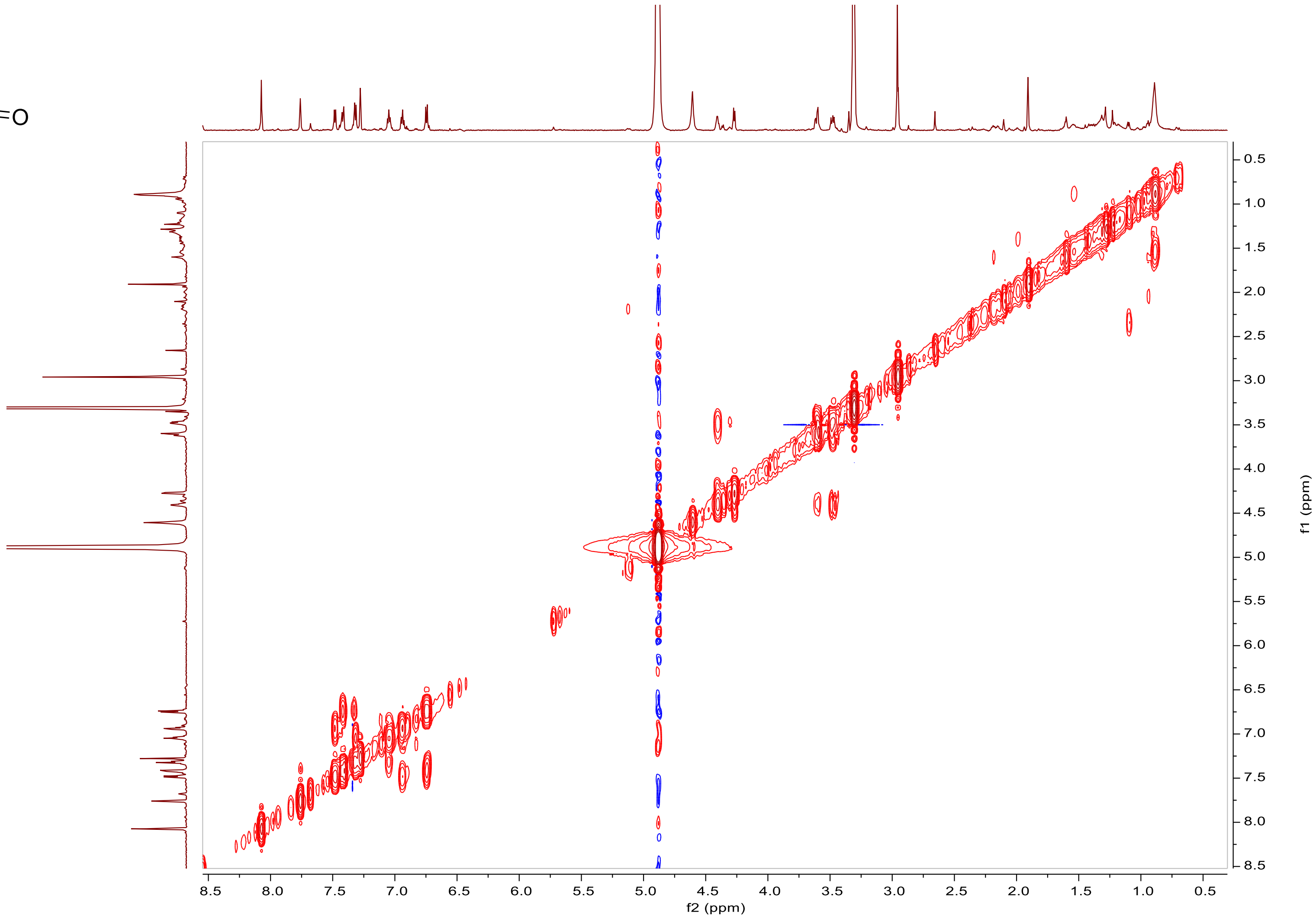

Figure S4. HSQC NMR Spectrum (CD_3OD, 700 MHz) of anithiactin D (**1**)

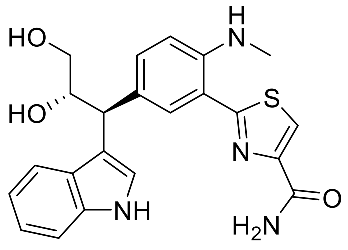

1

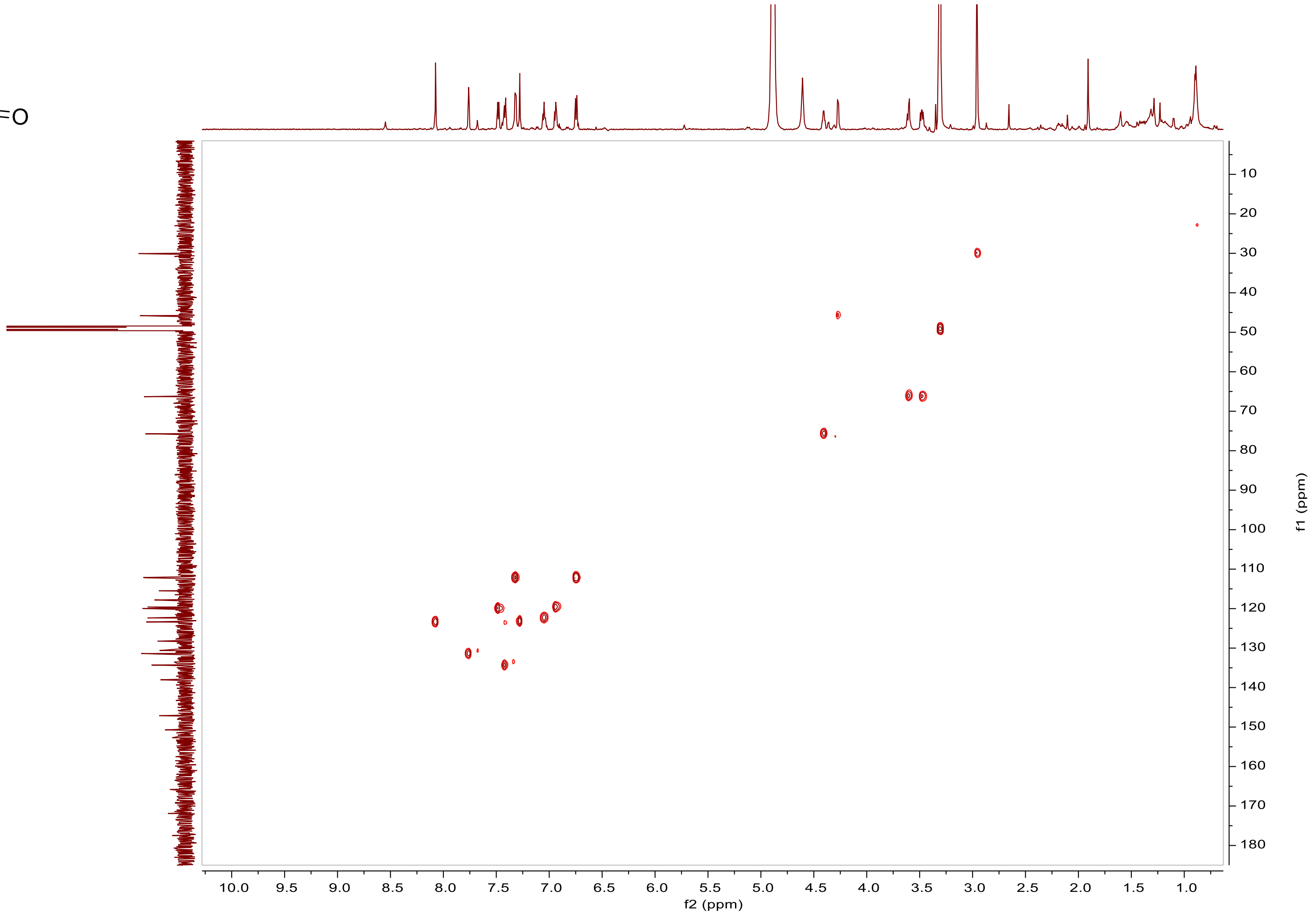

Figure S5. HMBC NMR Spectrum (CD_3OD, 700 MHz)of anithiactin D (**1**)

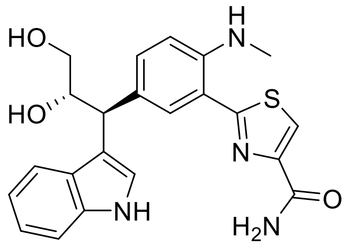

1

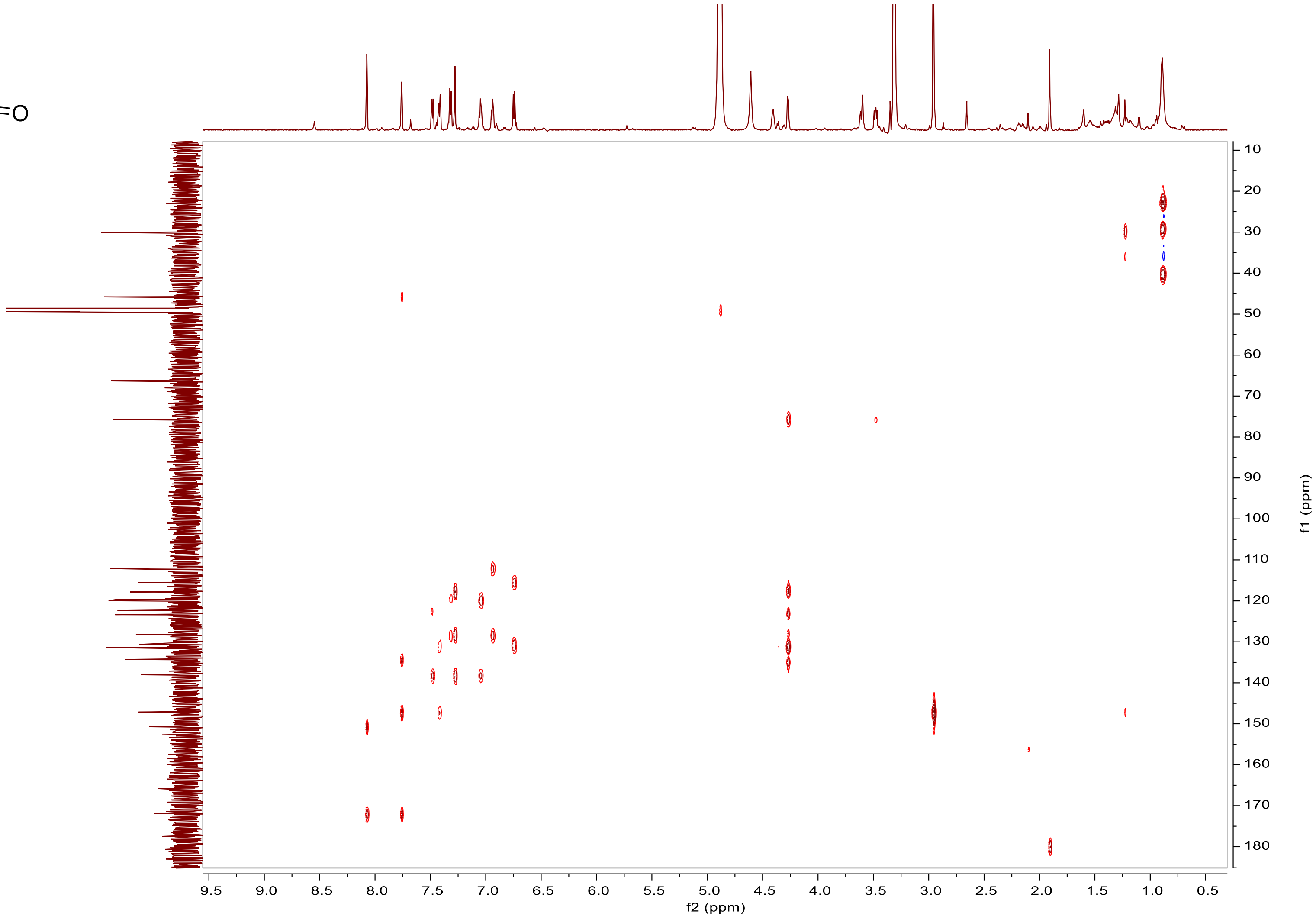

Figure S6. Comparing ECD spectrum with conformational search models of anthiactin D (**1**)

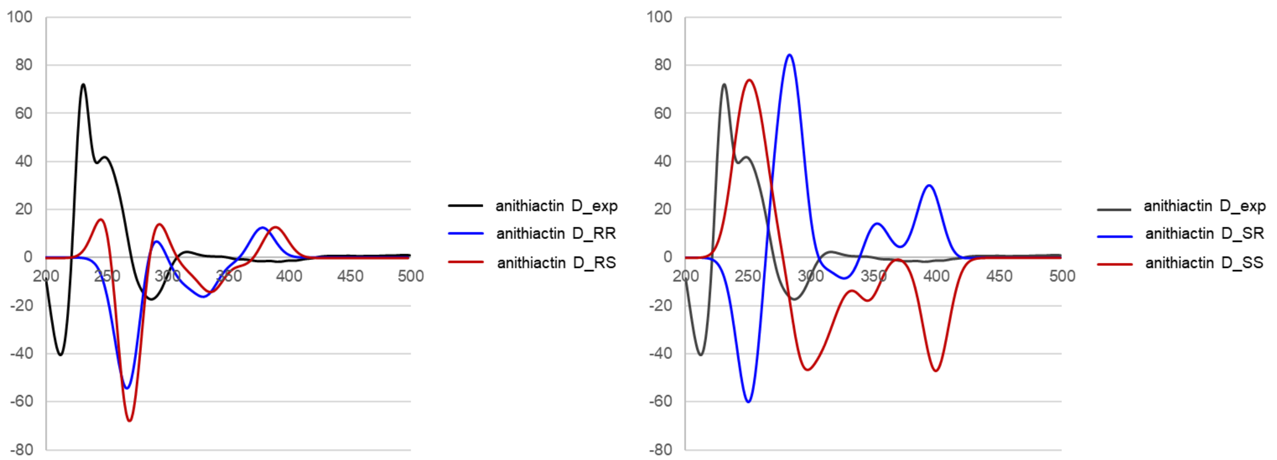

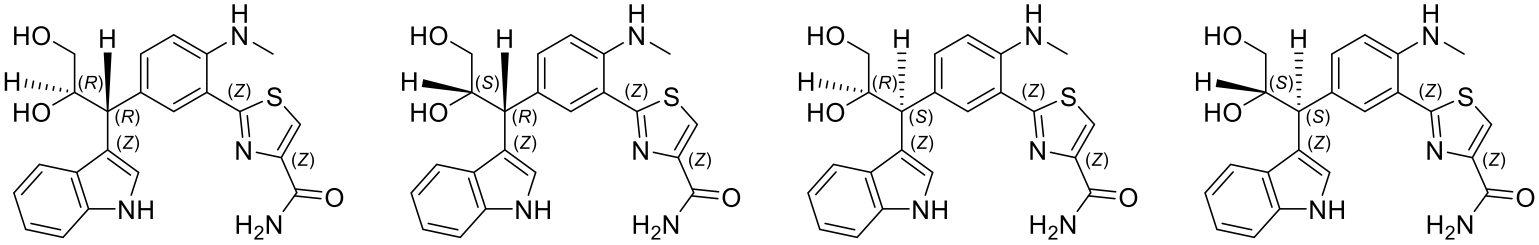

RR RS SR SS
